# Supplementary material for: Causal interplay between lactose intolerance and gut microbiota: a combined bidirectional Mendelian randomization and in vivo validation study
Source: Front Nutr. 2026 Jun 1;13:1803337. doi: 10.3389/fnut.2026.1803337 (PMC13265576; doi:10.3389/fnut.2026.1803337)
Supplement: Supplementary file 1 [file Data_Sheet_1.zip › supplementary materials/Forward/forest plot/ebi-a-GCST90027793.finngen_R12_E4_LACTONAS.pdf]

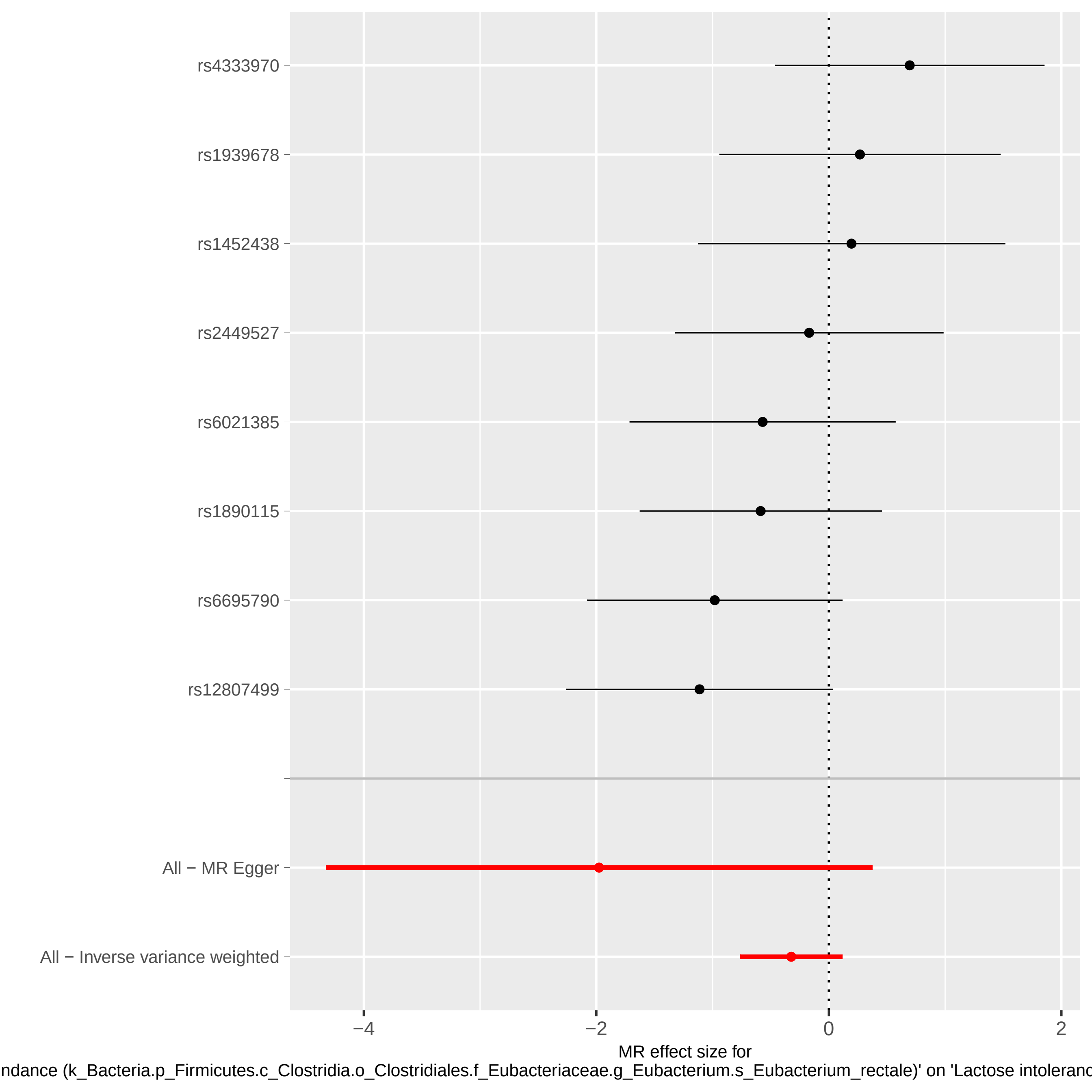

rs4333970
rs1939678
rs1452438
rs2449527
rs6021385
rs1890115
rs6695790
rs12807499
All − MR Egger
All − Inverse variance weighted
−4
−2
0
2
MR effect size for
ndance (k_Bacteria.p_Firmicutes.c_Clostridia.o_Clostridiales.f_Eubacteriaceae.g_Eubacterium.s_Eubacterium_rectale)' on 'Lactose intoleranc
